# Beyond VEGF: AEG-1/MTDH as a Systems-Level Orchestrator of Angiogenesis in Hepatocellular Carcinoma

**DOI:** 10.3390/cells15131214

**Published:** 2026-07-03

**Authors:** Rabha M. Younis, Kayla A. Rodriguez, Devanand Sarkar

**Affiliations:** 1 Department of Cellular, Molecular and Genetic Medicine, Virginia Commonwealth University, Richmond, VA 23298, USA; rabha.younis@vcuhealth.org (R.M.Y.); rodriguezka2@vcu.edu (K.A.R.); 2 Department of Cellular, Molecular and Genetic Medicine, Massey Comprehensive Cancer Center, Virginia Commonwealth University, Richmond, VA 23298, USA

**Keywords:** hepatocellular carcinoma (HCC), AEG-1/MTDH, angiogenesis, VEGF, hypoxia, therapeutic resistance, metabolic reprogramming

## Abstract

Hepatocellular carcinoma (HCC) remains one of the leading causes of cancer-related mortality worldwide and is characterized by extensive vascularization, aggressive progression, and limited therapeutic responsiveness. Angiogenesis plays a central role in HCC development by supporting tumor growth, metabolic adaptation, invasion, and metastatic dissemination. Although anti-angiogenic therapies targeting the vascular endothelial growth factor (VEGF) pathway have improved clinical management, their overall survival benefit remains modest because of compensatory signaling, adaptive resistance, and the highly complex nature of the tumor microenvironment (TME). Astrocyte elevated gene-1/metadherin (AEG-1/MTDH) has emerged as a multifunctional oncogene that functions by orchestrating interconnected angiogenic, inflammatory, metabolic, and immune-regulatory programs within the hepatic tumor microenvironment. AEG-1 regulates angiogenesis through modulation of VEGF-family signaling, NF-κB activation, hypoxia-responsive pathways, PI3K/AKT signaling, endothelial remodeling, and translational control of pro-angiogenic mediators. Emerging evidence further implicates AEG-1 in hypoxia adaptation, immune evasion, extracellular vesicle signaling, and metabolic reprogramming, supporting its role as a systems-level regulator of HCC angiogenesis. This review summarizes the current understanding of the molecular mechanisms through which AEG-1 regulates angiogenesis in HCC, discusses its interactions with the TME and anti-angiogenic resistance pathways, and highlights future translational opportunities for developing multi-targeted therapeutic strategies beyond conventional VEGF-centric approaches.

## 1. Introduction

### 1.1. Overview of the Global Burden and Clinical Challenges Associated with Hepatocellular Carcinoma (HCC)

Hepatocellular carcinoma (HCC), the most common form of primary liver cancer, is one of the leading causes of cancer-related mortality worldwide. HCC represents the sixth most common cancer globally and the third leading cause of cancer-related deaths [1,2]. Despite recent advances in therapeutic strategies, the disease remains highly aggressive and is characterized by a poor five-year survival rate of <30% [2,3]. Additionally, recurrence rates remain extremely high, reaching up to 88% [4]. The development of HCC is closely associated with chronic liver disease [5]. Persistent infections from hepatitis B virus (HBV) or hepatitis C virus (HCV) are well-established risk factors, as prolonged hepatic inflammation can promote the development of cirrhosis, in which healthy tissue is progressively replaced by fibrotic scar tissue [6]. Additional risk factors include excessive alcohol consumption, which contributes to cirrhosis, and metabolic dysfunction-associated steatohepatitis (MASH), an advanced form of metabolic dysfunction-associated fatty liver disease (MAFLD) [7,8,9]. Furthermore, obesity, diabetes, consumption of aflatoxin B1-contaminated food, and genetic conditions such as hereditary hemochromatosis, tyrosinemia, alpha-1 antitrypsin deficiency, glycogen storage diseases, porphyria cutanea tarda, and Wilson disease may increase the risk of developing HCC [7,10,11,12]. These conditions contribute to persistent liver injury, resulting in inflammation and fibrosis and cirrhosis, causing a tumor-promoting environment that can lead to HCC. A major clinical challenge in HCC is the difficulty of early detection [13,14]. HCC tends to develop silently, with most patients remaining asymptomatic during the early stages of disease. As a result, the majority of cases are diagnosed in the advanced stage, when curative treatments such as surgical resection or ablation are no longer viable [15]. As a result, late diagnosis remains one of the primary drivers of poor survival outcomes in HCC [16]. Even after curative treatment, HCC is associated with exceptionally high recurrence rates, which is driven by underlying chronic liver disease, creating a pro-tumorigenic environment that persists even after the primary tumor is removed [17]. Consequently, recurrence is common, often occurring within the first few years following treatment. Many patients in the advanced or recurrent stage of the disease rely on a combination of immunotherapy or tyrosine kinase inhibitors (TKIs). While the combination therapy provides a survival benefit of approximately two years, its efficacy is often limited by the unique biological landscape of the tumor [18]. A defining feature of HCC is its highly vascular nature, with tumor growth and progression heavily dependent on the formation of new blood vessels from the existing vasculature. Therefore, angiogenesis plays a crucial role in the development and metastasis of HCC.

### 1.2. Role of Angiogenesis in HCC Progression

Angiogenesis, the process of forming new blood vessels from pre-existing endothelial cells (ECs), is required for cancer cells to obtain nutrients and oxygen [19]. HCC relies on the continuous formation of new blood vessels to meet increasing metabolic and oxygen demands, allowing the lesion to grow beyond 1–2 mm^3^. This process is initiated by the angiogenic switch, where surrounding tissues produce pro-angiogenic growth factors, such as vascular endothelial growth factor (VEGF) and fibroblast growth factor (FGF). Hypoxia, defined as a state of insufficient oxygen supply to tissues, commonly arises in rapidly growing tumors and triggers angiogenesis by shifting the balance toward pro-angiogenic signaling [20]. Given the highly vascular nature of HCC, targeting angiogenesis has emerged as a promising therapeutic strategy.

### 1.3. Current Anti-Angiogenic Therapies and Their Clinical Limitations

Anti-angiogenic therapies have been widely adopted in the treatment of advanced HCC. Multiple agents targeting angiogenic signaling pathways have been developed and approved for clinical use; however, their overall impact on patient outcomes has been limited, with only modest extensions in survival. Sorafenib, the first TKI approved as a first line of treatment for advanced HCC, extends median overall survival by approximately 2–3 months compared to placebo [21]. Lenvatinib has been approved as a first-line treatment for advanced HCC, and demonstrated non-inferiority to sorafenib, with only a modest difference in median overall survival (13.6 vs. 12.3 months) [22]. As second-line treatment, Regorafenib has been approved for patients previously treated with sorafenib, and cabozantinib is used as an alternative second-line treatment. These second-line agents provide modest survival benefits, extending overall survival by only a few months [23,24]. A significant advance came with the combination of bevacizumab, a humanized monoclonal antibody targeting VEGF-A, and the immune checkpoint inhibitor (ICI) anti-programmed death ligand 1 (PD-L1) antibody, atezolizumab [25]. The landmark phase III IMbrave150 trial demonstrated superiority of this combination over sorafenib in prolonging both overall survival (hazard ratio (HR) = 0.58, 95% confidence interval (CI), 0.42–0.79) and progression-free survival (HR = 0.59, 95% CI 0.47–0.76) [25]. Despite the demonstrated prolonged survival with this combination treatment, this trial was interrupted after 8.6 months due to the median overall survival not being reached in patients treated with atezolizumab + bevacizumab. Recent efficacy and safety data has been published from the IMbrave150 trial, showing median overall survival of 19.2 months in the combination arm compared to 13 months in the sorafenib arm [26]. However, patients in the combination group had a higher incidence of serious adverse events as well as adverse events leading to discontinuation of treatment [25]. Commonly reported adverse events in the atezolizumab + bevacizumab arm included proteinuria, hypertension, aspartate, alanine aminotransferase increase, fatigue, pruritus, decreased appetite, and diarrhea [25,26]. Although this combination has proven to be the most effective FDA-approved first-line treatment for advanced unresectable HCC, the high number of serious adverse events presents a significant limitation.

Despite these advances, current anti-angiogenic therapeutic strategies are limited by only modest improvements in overall survival, the development of drug resistance, and the occurrence of adverse events. These challenges underscore the need to identify and target alternative molecular drivers of HCC progression.

### 1.4. Rationale for Investigating AEG-1/MTDH as a Critical Integrator of Angiogenic Pathways

Given the limitations of current therapeutic approaches, research has shifted toward identifying multi-functional scaffold proteins that can act as a critical integrator of angiogenic pathways. AEG-1 is overexpressed in nearly all cancers, including HCC, and plays a fundamental role in regulating the “angiogenic switch” [27,28,29,30]. While many pro-angiogenic factors trigger a single signaling cascade, AEG-1 acts as a central integrator, as it interacts with multiple transcription factors to simultaneously drive the expression of several pro-angiogenic factors [31]. This multifaceted role positions AEG-1 as a compelling therapeutic target and underscores the importance of further investigating its contribution to angiogenic regulation in HCC.

### 1.5. The Objectives of the Review: Presenting AEG-1 as a Systems-Level Regulator of Tumor Angiogenesis

This review aims to characterize AEG-1 not merely as an individual oncogene, but as a systems-level regulator that orchestrates the complex angiogenic microenvironment in HCC. By synthesizing current research, we aim to illustrate how AEG-1 integrates canonical pathways to drive an adaptive vascular phenotype. Furthermore, this review will examine the molecular structure of AEG-1 and its role in coordinating the intercellular crosstalk and microenvironmental factors that contribute to therapeutic resistance. Ultimately, by presenting AEG-1 as a central orchestrator of these diverse networks, we highlight its potential as a therapeutic target to overcome the limitations of current treatment models.

## 2. Biological Basis of Angiogenesis in HCC

### 2.1. Physiological Versus Pathological Angiogenesis in the Liver

Hepatic angiogenesis occurs in both physiological and pathological contexts. During liver regeneration, physiological angiogenesis supports the formation of new, functional sinusoidal vessels to restore blood flow. In contrast, pathological angiogenesis is marked by the development of capillarized vessels and intrahepatic shunts [32,33,34]. This aberrant intrahepatic vascular organization disrupts the normal interaction between sinusoids and hepatocytes, impairing oxygen and nutrient exchange. As a result, hepatic injury is exacerbated through increased inflammatory activity and intensified tissue hypoxia [33,34]. Pathological angiogenesis proceeds through two distinct mechanisms. Sprouting angiogenesis is considered the predominant pathway in neovascular tumors, including HCC. Sprouting angiogenesis, the formation of blood vessels from a preexisting capillary bed, is regulated by hypoxia-inducible factor-1α (HIF-1α), VEGF, and angiopoietin/Tie signaling [35,36]. The alternative mode of angiogenesis is intussusceptive angiogenesis, which involves the splitting of pre-existing vessels into two new vessels. Interestingly, intussusceptive angiogenesis has been shown to be stimulated by inhibition of sprouting angiogenesis in a rat model of HCC [37].

**Figure 1:**
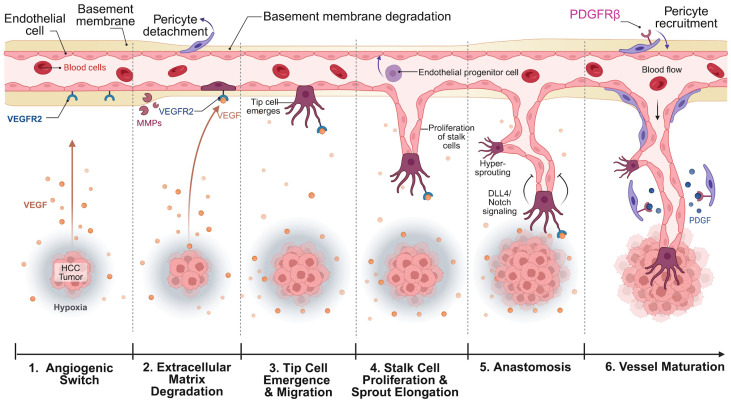
Stages of sprouting angiogenesis.

Angiogenesis is a multistep process initiated when the expanding tumor tissue triggers the angiogenic switch (Figure 1). In HCC, this switch is driven predominantly by hypoxia-induced upregulation of VEGF, which binds endothelial VEGF receptors (VEGFRs) to initiate motility and proliferation [38]. Activated ECs then produce matrix metalloproteinases (MMPs) that degrade the surrounding extracellular matrix (ECM) and basement membrane, creating space for vessel invasion. During this step, pericytes detach from the vessel wall to facilitate EC migration and tubulogenesis. Specialized tip cells emerge at the leading edge of the nascent sprout, extending filopodia and dactylopodia to sense and migrate along VEGF gradients, while trailing stalk cells proliferate to elongate the vessel [39,40]. During tube formation, endothelial progenitor cells intravasate into the blood vessels and through the bloodstream towards hypoxic areas, then extravasate and move into the surrounding tissue to help form the vessels’ inner lining [41]. Tip cell selection is governed by VEGF/Notch/delta-like canonical Notch ligand 4 (DLL4) signaling, which maintains the dynamic balance between the migratory tip cell phenotype and the proliferative stalk cell phenotype [42]. As adjacent sprouts extend, anastomosis, which is the fusion of tip cells from neighboring vessels, completes the vascular circuit and initiates blood flow. The new vasculature is initially immature and leaky, which allows cancer cells to metastasize to distant sites, but subsequent maturation is achieved through deposition of new ECM and recruitment of pericytes and smooth muscle cells by platelet-derived growth factor-β (PDGF-β), which stabilizes the vessel wall [43,44,45].

The expanding tumor triggers the angiogenic switch, driven by hypoxia-induced upregulation of VEGF, which binds to VEGFRs on endothelial cells to initiate motility and proliferation. Activated endothelial cells secrete MMPs that degrade the surrounding ECM and basement membrane, permitting vessel invasion, while pericytes detach from the vessel wall to facilitate endothelial cell migration. At the leading edge of the nascent sprout, specialized tip cells extend filopodia to sense and migrate along VEGF gradients, while stalk cells proliferate behind them to elongate the vessel. Tip-stalk cell specification is governed by VEGF/Notch/DLL4 signaling, which maintains the balance between the migratory tip cell and proliferative stalk cell phenotypes. During tube formation, endothelial progenitor cells are recruited through the bloodstream to hypoxic areas, where they extravasate and contribute to the vessel lining. Adjacent sprouts then undergo anastomosis, the fusion of tip cells from neighboring vessels, completing the vascular circuit and establishing blood flow. The newly formed vasculature is initially immature and leaky, facilitating metastatic dissemination, but subsequent maturation occurs through deposition of new ECM and recruitment of pericytes and smooth muscle cells via PDGF-β/PDGFRβ signaling, which stabilizes the vessel wall. DLL4, delta-like canonical Notch ligand 4; MMP, matrix metalloproteinase; PDGF-β, platelet-derived growth factor-β; PDGFRβ, platelet-derived growth factor receptor β; VEGF, vascular endothelial growth factor; VEGFR, vascular endothelial growth factor receptor. This was created with BioRender.

### 2.2. Influence of Hypoxia and Chronic Inflammation in Promoting Angiogenic Signals

Angiogenesis is driven by the combined effects of tissue hypoxia and chronic inflammation, which together create a microenvironment that favors sustained pro-angiogenic signaling. Hypoxia serves as a primary stimulus for angiogenesis through stabilization of hypoxia-inducible transcription factors. Under reduced oxygen conditions, HIF-1α accumulates and translocates to the nucleus, where it regulates the expression of various genes involved in angiogenic signaling, including VEGF [46]. In parallel, chronic inflammation further amplifies angiogenic responses by promoting the recruitment of immune cells, such as macrophages, monocytes, platelets, and mast cells [33]. These infiltrating cells contribute to the local production of angiogenic cytokines and growth factors, thereby promoting endothelial cell (EC) activation. Persistent inflammatory signaling promotes endothelial proliferation as well as migration, both of which are essential for new blood vessel formation. Chronic tissue injury and inflammation lead to prolonged endothelial activation, maintaining continuous pro-angiogenic signaling and vascular remodeling. These processes disrupt normal physiological angiogenesis and lead to both structural and functional hepatic disorganization. Accumulation of excess ECM, characterized by the replacement of physiological sinusoidal collagen (type IV) with fibrillar collagen (type I), alters hepatic sinusoidal architecture and results in the loss of endothelial fenestrations [32]. This fibrotic deposition evokes resistance to blood flow and reduces the delivery of oxygen to the liver parenchyma. The structural and functional impairment further reinforces hypoxic conditions, thereby sustaining pro-angiogenic signaling. Ultimately, this hostile environment of hypoxia and chronic inflammation serves as the primary driver for a complex interaction between malignant hepatocytes, ECs, and the surrounding stroma, driving the continuous crosstalk required to sustain pathological angiogenesis.

### 2.3. Intracellular Crosstalk Among Hepatocytes, Endothelial Cells (ECs) and Stromal Cells in the Angiogenic Site

Angiogenesis in HCC is regulated through coordinated interactions among multiple cell types within the hepatic microenvironment. This process depends on continuous communication between hepatocytes, ECs, and stromal cells through the exchange of cytokines, growth factors, and other signaling mediators. Crosstalk between hepatocytes and hepatic stellate cells (HSCs) generate a pro-angiogenic microenvironment in HCC, specifically by the induction of VEGFA and MMP9 expression in HSCs. Furthermore, gene expression patterns associated with this hepatocyte-HSC crosstalk correlate with HCC progression, poor prognosis, and metastasis propensity in human HCCs [47]. Additionally, HSCs alone function as liver-specific pericytes. HSCs exhibit pro-angiogenic activity upon activation, including the production of VEGF and Angiopoietin-1 (Ang-1) and their receptors in hypoxic circumstances [48,49]. Activated HSCs can also promote the recruitment of inflammatory cells, which further sustains the angiogenic process [50]. In addition, liver-resident macrophages, Kupffer cells (KC) and other immune populations contribute to angiogenesis through the release of cytokines, reactive oxygen species (ROS), and platelet-activating factors (PAFs), which activate signaling pathways, such as nuclear factor kappa light chain enhancer of activated B cells (NF-κB), and enhance the expression of pro-angiogenic mediators, such as VEGF [34,51,52,53]. Together, these interconnected cellular interactions establish a dynamic network that sustains angiogenic signaling within the liver.

## 3. Canonical Angiogenic Pathways in HCC

### 3.1. Overview of Vascular Endothelial Growth Factor (VEGF) and Its Receptors (VEGFRs)

The VEGF family represents a central regulator of EC function and plays a critical role in both physiological and pathological angiogenesis. The VEGF family consists of several ligands, including VEGF-A, VEGF-B, VEGF-C, VEGF-D, and VEGF-E, which collectively contribute to angiogenesis [54,55]. In the context of HCC, elevated VEGF expression is strongly associated with tumor aggressiveness, promoting enhanced tumor growth, vascularization, and metastatic potential via promoting angiogenesis [56]. Increased circulating VEGF levels have also been observed in patients with HCC and are correlated with greater tumor microvessel density as well as poor prognosis [57]. VEGF exerts its biological effects through binding to specific tyrosine kinase receptors, primarily VEGFR1, VEGFR2, and VEGFR3. Interactions between VEGF ligands and these receptors activate signaling pathways that regulate EC behavior [58]. VEGFR2, in particular, is widely expressed on ECs and serves as the principal mediator of angiogenic signaling [58]. Upon ligand binding, especially VEGF-A, VEGFR2 initiates intracellular signaling cascades, including the phosphatidylinositol 3-kinase (PI3K)/AKT and RAF/mitogen activated protein kinase (MAPK) pathways, which drive EC proliferation, migration, and the formation of new vascular structures [56,58]. While VEGFR1 contributes to vascular development through ligand binding, VEGFR3 is more specifically involved in lymphangiogenesis through its interactions with VEGF-C and VEGF-D [56]. In addition to promoting vessel formation, VEGF signaling increases vascular permeability, which may lead to the development of structurally abnormal and leaky vessels. This altered vasculature contributes to elevated interstitial pressure and the formation of hypoxic or necrotic regions within tumors, which can further stimulate angiogenesis and support tumor progression. Together, these findings highlight the VEGF/VEGFR axis as a principal mediator of angiogenic processes in HCC. 

### 3.2. Angiopoietin–Tie Receptor Axis

The angiopoietin–Tie signaling pathway represents another key regulatory system involved in angiogenesis, particularly in the context of vascular remodeling and stabilization. Ang-1 and angiopoietin-2 (Ang-2) are the primary ligands for the endothelial-specific tyrosine kinase receptor Tie2. Although these ligands share structural similarities and bind Tie2 with comparable binding affinity, they exert distinct and often opposing biological effects on vascular function. Ang-1 is broadly expressed in adult tissues and plays a central role in maintaining vascular integrity. It promotes the maturation and stabilization of newly formed blood vessels by strengthening EC junctions and facilitating the recruitment of pericytes and smooth muscle cells [59,60]. In contrast, Ang-2 is more prominently associated with vascular remodeling [61,62]. Its expression is upregulated in pathological conditions, including liver cirrhosis and HCC, where it contributes to disease progression [63]. Notably, Ang-2 levels increase with advancing tumor stage and have been associated with poorer clinical outcomes [64]. Functionally, Ang-2 can act as a context-dependent regulator of angiogenesis. In the absence of VEGF, Ang-2 disrupts vascular stability and promotes vessel regression by counteracting the stabilizing effects of Ang-1. However, in the presence of VEGF signaling, Ang-2 enhances EC proliferation and migration, thereby facilitating angiogenic growth [65]. In HCC, co-overexpression of Ang-2 and VEGF has been shown to accelerate tumor progression and angiogenesis while reducing vessel maturation and intratumoral apoptosis, suggesting a synergistic interaction between these pathways [66]. Emerging evidence also suggests that Ang-2 expression is influenced by the TME [67]. While its levels are not significantly elevated in HCC tumor cells themselves, ECs can release Ang-2 under hypoxic conditions [56]. Additionally, hypoxia-induced VEGF signaling may upregulate Ang-2 expression, reinforcing angiogenic activity [68]. These findings highlight the importance of the Ang/Tie axis, particularly Ang-2, as a dynamic regulator of angiogenesis.

### 3.3. Platelet-Derived Growth Factor (PDGF), Fibroblast Growth Factor (FGF) and Hepatocyte Growth Factor (HGF)-Mediated Angiogenesis

In addition to VEGF and angiopoietin signaling, several other growth factor pathways contribute to the regulation of angiogenesis in HCC, including PDGF, FGF, and HGF. These factors act through distinct yet interconnected mechanisms to promote EC activation, vascular remodeling, and tumor progression. PDGFs comprise a family of dimeric growth factors encoded by multiple genes and are known to regulate the proliferation and migration of glial cells and vascular-associated cells, including fibroblasts and smooth muscle cells [56,69]. In HCC, both PDGFs and their receptors are frequently upregulated and have been associated with poor prognosis and shorter overall survival when co-expressed with VEGFR-3 [70]. Activation of PDGF signaling engages downstream pathways such as PI3K/AKT and MAPK/extracellular signal-regulated kinase (ERK), which contribute to both tumor cell proliferation and angiogenesis [71]. Elevated expression of PDGF receptors has also been correlated with increased microvessel density and poorer prognosis [72,73,74]. These findings establish PDGF signaling as a key contributor to the proangiogenic phenotype of HCC. FGFs represent another important class of pro-angiogenic mediators, consisting of a large family of ligands involved in cell proliferation and angiogenesis. Several cancers, including HCC, may be induced by abnormal FGF/FGFR signaling [75,76,77,78,79]. Through interaction with its receptor, FGFR1, FGF2 activates downstream signaling pathways such as RAF/MAPK, leading to EC proliferation and angiogenic progression [80]. Beyond its direct effects on ECs, FGF2 also enhances recruitment of host cells to the TME and cooperates with VEGF to promote neovascularization [81,82]. This interaction contributes to structural changes such as sinusoidal capillarization and supports tumor growth and metastasis [83]. The importance of FGF signaling is further underscored by studies demonstrating that inhibition of FGF2 activity can suppress both tumor growth and angiogenic signaling [84,85]. Lastly, HGF promotes angiogenesis through activation of its receptor, Met, which contributes to angiogenesis by regulating both pro- and anti-angiogenic factors. It has been shown to increase the expression of VEGF, a major promoter of angiogenesis, while concurrently suppressing thrombospondin-1 (TSP-1), a negative regulator of angiogenesis [86]. This coordinated regulation shifts the balance toward a pro-angiogenic state, supporting tumor development. VEGF induction by HGF involves multiple signaling pathways, including MAPK, PI3K, and signal transducer and activator of transcription 3 (STAT3), whereas the suppression of TSP-1 appears to depend primarily on MAPK signaling [86]. Although these findings are not specific to HCC, they provide insight into how HGF/Met signaling functions as a key regulator of the angiogenic switch in tumor progression. Collectively, PDGF, FGF, and HGF signaling pathways contribute to angiogenesis through complementary mechanisms that promote EC activation, vascular remodeling, and tumor progression. Although these growth factors operate through distinct receptors and pathways, they converge on shared pro-angiogenic outcomes within the TME. Their activity is further amplified by hypoxia, which stimulates pro-angiogenic signaling. Central to this process are hypoxia-inducible factors (HIFs), which regulate angiogenic gene expression and play a critical role in HCC vascularization.

### 3.4. Hypoxia-Inducible Factors (HIF-1α/HIF-2α) and Their Regulatory Roles

HIFs play a central role in HCC progression, with clinical studies consistently demonstrating elevated expression of HIF-1α in HCC tissues [60,87,88,89,90,91,92,93,94]. Increased HIF-1α expression is strongly associated with poor prognosis, vascular proliferation, recurrence, and metastasis [48,89,90]. In contrast, HIF-2α expression shows a more variable relationship with clinical outcomes, suggesting distinct regulatory roles between HIF-1α and HIF-2α in liver cancer progression [87,95]. A key function of HIF signaling in HCC is the regulation of tumor angiogenesis. Under hypoxic conditions, HIF-1α acts as a major transcriptional driver of pro-angiogenic gene expression, most notably VEGF [96]. HIF-1α enhances VEGF expression through transcriptional activation involving binding to hypoxia response elements while also increasing VEGF mRNA stability, thereby amplifying angiogenic signaling [97]. Experimental evidence highlights its importance, as inhibition of HIF-1α activity leads to significantly delayed HCC development [98]. Beyond VEGF, HIF-1α regulates a broader angiogenic program in HCC, including PDGF, transforming growth factor (TGF), angiopoietins, erythropoietin (EPO), and fibroblast growth factors, (FGF), all of which contribute to angiogenesis in tumors [96,99,100,101]. HIF-2α also contributes to angiogenesis in HCC, primarily through regulation of VEGF and additional angiogenic mediators such as erythropoietin, VEGFR2, angiogenin, and Tie-2, further supporting tumor vascular development in HCC [94,102,103]. Together, HIF-1α and HIF-2α function as key regulators of hypoxia-driven angiogenesis in HCC through coordinated activation of pro-angiogenic signaling pathways.

### 3.5. Limitations of Pathway-Specific Inhibition in Clinical Settings and Rationale for Integrative Angiogenic Regulators Such as AEG-1

Despite substantial progress in identifying key pro-angiogenic signaling pathways in HCC, therapeutic strategies targeting individual pathways have demonstrated limited clinical efficacy. A major challenge of pathway-specific inhibition is the compensatory crosstalk that exists among angiogenic networks [104]. Suppression of a single pathway, such as VEGF signaling, may reduce angiogenic activity initially, but parallel pathways can sustain vascular remodeling and restore pro-angiogenic signaling. This adaptive plasticity contributes to therapeutic resistance and helps explain the limited durability of current anti-angiogenic approaches in HCC. These limitations highlight the need to investigate broader upstream regulators capable of coordinating multiple angiogenic programs simultaneously, thereby providing a stronger rationale for focusing on integrative modulators such as AEG-1.

## 4. AEG-1/MTDH: Structure, Function and Regulation

### 4.1. Discovery and Nomenclature: AEG-1/MTDH/LYRIC

AEG-1 was initially identified and cloned in primary human fetal astrocytes (PHFAs) in 2002 through rapid subtraction hybridization (RaSH) as an HIV-1-, gp120- and tumor necrosis factor α (TNFα)-inducible gene [105]. In 2004, the mouse homolog of AEG-1 was cloned as MTDH and identified as a cell membrane protein involved in mediating breast cancer metastasis to the lungs through an in vivo phage screening approach [27]. In the same year, gene trapping techniques enabled the cloning of the rodent (mouse/rat) homolog, termed lysine-rich CEACAM-1 co-isolated protein (LYRIC), which was characterized as an endoplasmic reticulum (ER)/nuclear envelope-associated protein as well as a tight junction protein [106,107]. Since its discovery, extensive studies have revealed that AEG-1 is frequently overexpressed in numerous cancers, including HCC, where its expression has been associated with tumor progression, metastasis, angiogenesis, and poor clinical outcome [108,109,110,111,112,113]. Collectively, these studies established AEG-1 as an oncogene involved in coordinating several signaling pathways associated with HCC progression.

### 4.2. Protein Structure, Localization and Interaction Networks

**Figure 2:**
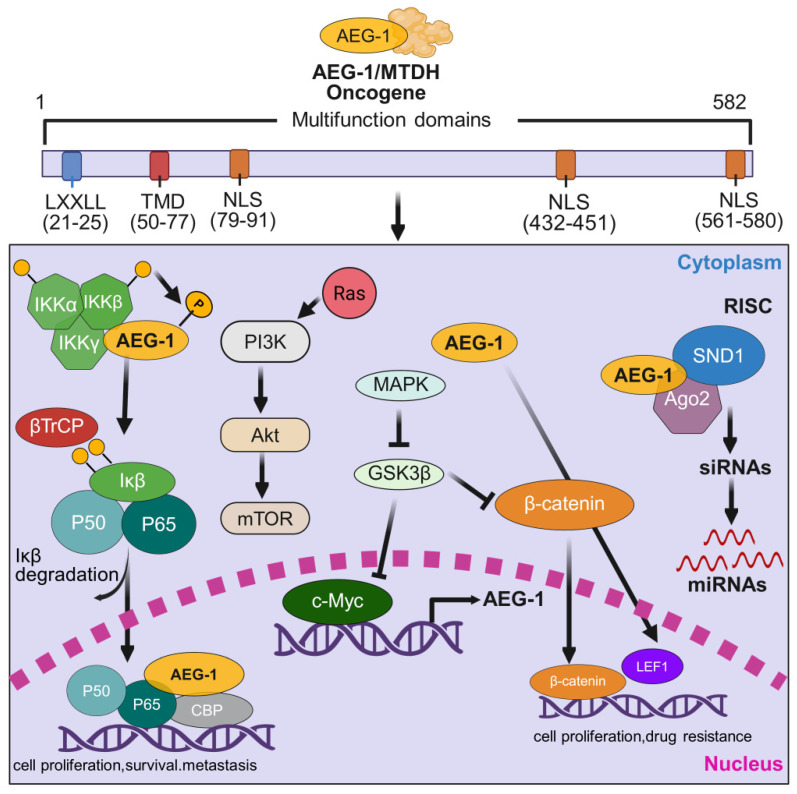
Schematic representation of the structure and oncogenic functions of Astrocyte Elevated Gene-1 (AEG-1), also known as Metadherin (MTDH). The upper panel illustrates the domain or-ganization of the 582-amino-acid AEG-1 protein, including the LXXLL motif (aa 21–25), trans-membrane domain (TMD; aa 50–77), and three nuclear localization signals (NLSs; aa 79–91, 432–451, and 561–580). The lower panel summarizes the major signaling pathways regulated by AEG-1 in cancer. AEG-1 activates the NF-κB pathway through interaction with the IKK complex, pro-moting IκB degradation, nuclear translocation of p50/p65, and transcription of genes involved in cell proliferation, survival, and metastasis. AEG-1 also enhances PI3K/Akt/mTOR signaling, con-tributing to tumor growth and survival. Through modulation of MAPK/GSK3β signaling, AEG-1 stabilizes c-Myc and β-catenin, leading to increased transcriptional activity of oncogenic target genes. In the nucleus, AEG-1 cooperates with β-catenin/LEF1 and transcriptional coactivators such as CBP to promote gene expression associated with proliferation and drug resistance. Additionally, AEG-1 interacts with SND1 and Argonaute-2 (Ago2) within the RNA-induced silencing complex (RISC), enhancing miRNA-mediated gene silencing. Collectively, these pathways establish AEG-1/MTDH as a multifunctional oncogene that drives tumor initiation, progression, metastasis, and therapeutic resistance. AEG-1, astrocyte elevated gene-1; MTDH, metadherin; LXXLL, leucine-X-X-leucine-leucine motif; TMD, transmembrane domain; NLS, nuclear localization signal; IKK, IκB kinase; IκB, inhibitor of NF-κB; NF-κB, nuclear factor kappa B; βTrCP, beta-transducin repeat-containing protein; PI3K, phosphoinositide 3-kinase; Akt, protein kinase B; mTOR, mecha-nistic target of rapamycin; MAPK, mitogen-activated protein kinase; GSK3β, glycogen synthase kinase 3 beta; CBP, CREB-binding protein; LEF1, lymphoid enhancer-binding factor 1; RISC, RNA-induced silencing complex; SND1, staphylococcal nuclease and Tudor domain-containing protein 1; Ago2, Argonaute 2; siRNA, small interfering RNA; miRNA, microRNA. Created with BioRender.

AEG-1 is a highly conserved lysine-rich protein composed of 582 amino acids in humans and is predominantly expressed in vertebrates [114,115]. Because AEG-1 is a single-pass transmembrane protein, the complete three-dimensional structure of AEG-1 has not yet been resolved because of technical difficulties of crystalizing membrane proteins. However, protein interactome coupled with deletion mutation analyses have identified multiple functional motifs and interaction regions that provide insight into its biological activity. AEG-1 contains a transmembrane domain (TMD) between amino acid residues 50–77 that facilitates anchoring to the ER membrane and, in aggressive tumor cells, the cell membrane [27,114,116,117]. In addition, three lysine-rich nuclear localization signals (NLSs), located within residues 79–91, 432–451, and 561–580, regulate nuclear and nucleolar localization [118,119] (Figure 2). Beyond its localization patterns, AEG-1 participates in numerous protein–protein and protein–RNA interactions that contribute to its diverse cellular functions. Although AEG-1 lacks RNA-binding domains, its intrinsically disordered central region mediates RNA-binding activity and contributes to translational regulation [120]. AEG-1 also interacts with components of the NF-κB signaling pathway, including K63-linked ubiquitinated proteins such as TNF receptor associated factor 2 (TRAF2) and receptor interacting serine/threonine kinase 1 (RIPK1), thereby facilitating NF-κB activation [120]. Additional studies demonstrated that inhibitor of nuclear factor kappa B kinase subunit beta (IKKβ)-mediated phosphorylation of AEG-1 promotes IκBα degradation and NF-κB nuclear signaling, further supporting its role in inflammatory and pro-survival signaling pathways [120]. Several additional motifs contribute to the pleiotropic functions of AEG-1. An LXXLL motif located near the N-terminus mediates interaction with retinoid X receptor (RXR), allowing AEG-1 to negatively regulate its activity [120]. A lung-homing domain spanning residues 381–442 has been implicated in metastatic adhesion to pulmonary endothelium which contributes to the metastasis of these cells to the lungs [118,119]. Importantly, one of the strongest characterized AEG-1 interactions occurs with staphylococcal nuclease and tudor domain-containing 1 (SND1), a multifunctional RNA-binding protein involved in oncogenic RNA processing [121,122,123]. Structural studies identified residues W394 and W401 as critical for the AEG-1/SND1 interaction [124,125,126,127]. Residue cysteine 75 (Cys75) of AEG-1 is modified by palmitoylation, a post-translational modification in which fatty acids are covalently attached to cysteine residues, by zDHHC palmitoyltransferase 6 (ZDHHC6) [128,129,130]. Palmitoylation negatively regulates AEG-1 function, which was documented by studying a knock-in mouse in which AEG-1 Cys75 was mutated to either serine or alanine [128,129,130].

### 4.3. Regulation of AEG-1 Expression in HCC

AEG-1 expression in HCC is regulated by multiple tumor-promoting stimuli within the hepatic microenvironment, including hypoxic stress, inflammatory signaling, and dysregulated oncogenic pathways (Figure 2). These mechanisms sustain AEG-1 overexpression during hepatocarcinogenesis and promote tumor progression.

#### Hypoxia and Inflammatory Signaling: Role of PI3K/AKT and NF-κB Pathways

Hypoxia is a defining feature of the HCC tumor microenvironment and arises from rapid tumor growth that exceeds the capacity of the existing vasculature. This hypoxic stress functions as an important regulatory cue that alters the AEG-1 expression. In HCC, exposure to hypoxic conditions has been shown to increase AEG-1 expression at both the mRNA and protein levels, indicating that oxygen availability directly influences AEG-1 regulation [131]. Hypoxia-driven regulation of AEG-1 is closely linked to the activation of oxygen-sensing signaling pathways, particularly those involving HIF-1α and PI3K/AKT signaling [131,132]. Hypoxia activates PI3K/AKT signaling, which contributes to stabilization and activation of HIF-1α [131,132]. AEG-1 has been identified as a downstream effector of this signaling network, suggesting that hypoxic conditions promote AEG-1 upregulation through PI3K/AKT signaling [131]. Conversely, inhibition of AEG-1 disrupts this signaling balance [131]. Together, these findings support a model in which hypoxic stress within the HCC microenvironment contributes to AEG-1 overexpression through activation of hypoxia-adaptive signaling pathways. The PI3K/AKT pathway is frequently dysregulated in HCC and represents a major upstream regulator of AEG-1 expression [133,134]. Mechanistic studies demonstrated that oncogenic Ha-ras induces AEG-1 transcription through PI3K-dependent signaling pathways [135]. Activation of PI3K/AKT signaling increased c-Myc activity and promoted c-Myc binding to E-box elements within the AEG-1 promoter, resulting in enhanced AEG-1 transcription [135]. Inhibition of PI3K signaling or restoration of phosphatase and tensin homolog (PTEN) activity significantly attenuated AEG-1 promoter activation, further supporting the role of PI3K/AKT signaling in regulating AEG-1 expression [135]. AEG-1 can also activate the PI3K/AKT pathway, contributing to tumorigenesis [136]. These findings suggest that aberrant PI3K/AKT signaling contributes to sustained AEG-1 overexpression in HCC, while AEG-1 in turn sustains PI3K/AKT activity.

In addition to hypoxia, chronic inflammation is a defining feature of the HCC microenvironment and plays a major role in regulating AEG-1 expression. Pro-inflammatory cytokines, including TNF-α and IL-1β, have been shown to induce AEG-1 expression through activation of NF-κB signaling pathways [137,138,139]. Inflammatory stimulation, such as TNF-α, also enhances the nuclear activity of AEG-1, where it interacts with the p65 subunit of NF-κB and the transcriptional co-activator CREB-binding protein (CBP) to promote NF-κB signaling [140,141]. As mentioned earlier, AEG-1 interacts with upstream regulators of NF-κB facilitating its activation [27,142]. Taken together, these findings demonstrate that AEG-1 and NF-κB engage in a regulatory loop, in which inflammatory signals induce AEG-1 expression while AEG-1 amplifies NF-κB activity to sustain pro-tumorigenic signaling in HCC.

### 4.4. AEG-1 Cooperativity with Oncogenic Partners in HCC Progression

#### 4.4.1. Cooperation of AEG-1 and c-Myc

AEG-1 drives HCC progression through interactions with key oncogenic drivers. A well-characterized interaction exists between AEG-1 and c-Myc, a major driver oncogene in HCC encoded by MYC [143]. MYC directly regulates AEG-1 transcription via binding to its promoter, establishing AEG-1 as a transcriptional target of c-Myc signaling [135]. Conversely, AEG-1 enhances c-Myc expression and activity through various mechanisms, including repression of transcriptional inhibitors such as zinc finger and BTB domain containing 16 (ZBTB16/PLZF) and upstream pathways such as Wnt/β-catenin signaling, which further contributes to MYC upregulation [28,144]. These reciprocal regulatory interactions establish a feedforward loop in which c-Myc promotes AEG-1 expression, while AEG-1 reinforces c-Myc-driven transcription. Genomic evidence further supports this relationship, as both AEG-1 and MYC are located on chromosome 8q and are frequently co-amplified in a subset of HCC cases in The Cancer Genome Atlas (TCGA). Dual overexpression of AEG-1 and c-Myc in mouse hepatocytes (Alb/AEG-1/c-Myc mouse) results in spontaneous development of metastatic HCC, as well as marked increase in tumor burden in response to the hepatocarcinogen N-nitrosodiethylamine (DEN), compared to mice expressing each gene alone (Alb/AEG-1 or Alb/c-Myc) [145]. Additionally, Alb/AEG-1/c-Myc hepatocytes showed enhanced proliferation, invasion and chemoresistance compared to Alb/AEG-1 or Alb/c-Myc hepatocytes [145]. RNA sequencing analyses of the livers revealed that combined AEG-1 and c-Myc expression induces a distinct gene signature in non-coding RNAs that are required for tumor progression, underscoring their cooperative role in shaping an aggressive HCC phenotype [145].

#### 4.4.2. Interaction Between AEG-1 and SND1

Another major mechanism underlying AEG-1 oncogenic function in HCC involves its interaction with SND1. SND1, also known as p100 activator or Tudor staphylococcal nuclease (Tudor-SN) was identified as the strongest AEG-1 interacting protein through yeast two-hybrid screening and co-immunoprecipitation using a human liver complementary DNA (cDNA) coupled with mass spectrometry approaches [121,122]. SND1 is localized in the nucleus and cytoplasm, where it regulates various cellular processes, including transcription, RNA splicing, and RNA metabolism [146,147,148,149]. In the cytoplasm, SND1 and AEG-1 co-operate within the RNA-induced silencing complex (RISC), where they facilitate gene silencing by small RNAs, such as siRNA or miRNA [121,150]. This interaction promotes the degradation of tumor suppressor mRNAs, including PTEN, a target of miR-221 that is overexpressed in HCC [121]. Consistent with this function, increased expression of either AEG-1 or SND1 enhances RISC activity [121]. Functionally, the AEG-1-SND1 axis has been directly linked to hepatocarcinogenesis [151,152]. SND1 is overexpressed in HCC and promotes tumor growth in xenograft and transgenic models, while hepatocyte specific SND1 overexpression enhances both spontaneous and DEN-induced HCC [121,152]. Upon DNA replication stress induction, SND1 protein half-life is significantly reduced when AEG-1 is knocked down, indicating that interactions between SND1 and AEG-1 are necessary for survival under stressful conditions [123]. Additionally, AEG-1 enhances SND1 protein stability under cellular stress conditions such as heat shock [124]. In a clear cell renal cell carcinoma model, disruption of AEG-1-SND1 interaction resulted in lack of tumorigenic capacity, suggesting that this interaction may be required for additional tumorigenesis [153]. 

#### 4.4.3. Feedback Loop Between AEG-1 and Wnt/β-catenin Signaling

Studies have demonstrated that AEG-1 closely interacts with the Wnt/β-catenin pathway in HCC [154,155]. AEG-1 overexpression alters the expression of multiple components of the Wnt/β-catenin signaling cascade, including induction of lymphoid enhancer-binding factor (LEF1) and its downstream target MYC [28]. In addition, AEG-1 suppresses negative regulators of Wnt signaling such as adenomatous polyposis coli (APC) and C-terminal binding protein 2 (CTBP2), while promoting ERK-mediated phosphorylation and degradation of glycogen synthase kinase β (GSK3β), thereby facilitating β-catenin nuclear translocation [28]. Functional studies further demonstrated that LEF1 knockdown significantly inhibited AEG-1-induced HCC cell proliferation and invasion, supporting the importance of the Wnt/β-catenin signaling in mediating AEG-1 [156]. These studies highlight the regulatory crosstalk between AEG-1 and the Wnt/β-catenin pathway, where AEG-1 modulates multiple components of the Wnt signaling cascade to promote HCC progression. A direct interaction between AEG-1 and β-catenin was observed in colorectal cancer cells [157]. The cooperative interactions between AEG-1 and its oncogenic partners, c-Myc, SND1, and the Wnt/β-catenin pathway, establish an interconnected signaling network that amplifies hepatocarcinogenesis and underscores AEG-1’s role as a central orchestrator of HCC progression.

## 5. AEG-1 as a Central Regulator of Angiogenesis in HCC

### 5.1. Transcriptional Regulation and Epigenetic Control

The clearest evidence linking AEG-1 to angiogenesis in HCC involves the VEGF signaling pathway. HepG3 cells overexpressing AEG-1 produced significantly higher levels of VEGF, placental growth factor (PlGF), and FGFα compared with control cells [109]. Xenograft tumors derived from these cells also showed increased vascularization. Similarly, conditioned medium from Alb/AEG-1 hepatocytes induced differentiation of human vascular endothelial cells (HUVECs) [145]. These findings indicated that AEG-1 regulates a broader angiogenesis-related transcriptional network in HCC, leading subsequent studies to recognize AEG-1 as an important pro-angiogenic factor in liver cancer rather than solely a promoter of metastasis. In several angiogenesis models, increased Ang-1 expression occurred alongside elevated HIF-1α and additional vascular markers, supporting the possibility that AEG-1 contributes to angiopoietin signaling. Research has shown that AEG-1 can interact with NF-κB at the interleukin-8 (IL-8) promoter, functioning as a transcriptional coactivator to enhance IL-8 expression [141]. It was demonstrated that AEG-1-interacting protein SND1 promotes angiogenesis in HCC through an NF-κB/miR-221/angiogenin-CXCL16 signaling cascade [151]. Thus, AEG-1/SND1 interaction results in a broad spectrum of angiogenesis induction.

Evidence supporting AEG-1’s role in regulating the HIF-1α axis is compelling, although many of the earliest mechanistic studies were conducted outside the context of liver cancer. In both HUVEC and glioma models, AEG-1 increases HIF-1α levels, whereas inhibition of Akt signaling suppresses AEG-1-mediated HIF-1α induction and the associated pro-angiogenic endothelial responses [108]. Within HCC, AEG-1 has been implicated in hypoxia-driven chemoresistance via the PI3K/Akt/HIF-1α/MDR1 signaling cascade [108]. Although this finding does not directly establish an angiogenic role, it demonstrates that AEG-1 reinforces the core hypoxia-response network that commonly promotes angiogenic signaling in liver tumors. Given the established role of HIF-1α in regulating VEGF-mediated neovascularization in HCC, these observations remain highly relevant to discussions of tumor angiogenesis.

An important HCC-specific example of post-transcriptional regulation emerged from hepatocyte-specific Alb/AEG-1 transgenic model. Conditioned medium derived from Alb/AEG-1 hepatocytes exhibited strong pro-angiogenic activity which was mediated by coagulation factor XII. It was demonstrated that AEG-1 enhanced factor XII protein production by promoting polysome loading of its mRNA [111]. These findings suggest that AEG-1 can modify the angiogenic secretory profile of HCC cells through selective translational regulation, independent of direct promoter activation alone. Additional complexity may arise from post-translational modification of AEG-1 itself. For example, palmitoylation has been shown to alter AEG-1/SND1 signaling behavior and influence HCC progression, although current evidence has not conclusively demonstrated that this modification directly regulates angiogenic output [128,129,130]. 

### 5.2. AEG-1 as a Modulator of Hypoxia Signaling

#### 5.2.1. AEG-1’s Role in Stabilizing and Amplifying HIF Responses

AEG-1 amplifies HIF-associated signaling under low-oxygen conditions. Low oxygen tension is a frequent feature of HCC and has been shown experimentally to elevate AEG-1 levels in HCC models. In HepG2 cells, hypoxic exposure led to increased expression of both AEG-1 and MDR-1 relative to normoxic conditions. Silencing AEG-1 enhanced apoptosis triggered by chemotherapeutic agents under hypoxia and reduced activity along the PI3K/AKT/HIF-1/MDR-1 axis [131]. Although the primary focus of this study is drug resistance, the signaling pathways involved are also closely tied to angiogenesis. Specifically, PI3K/AKT and HIF-1 pathways regulate the production of VEGF and other pro-angiogenic factors. This positions AEG-1 as a key intermediary that responds to hypoxia and helps maintain signaling cascades responsible for angiogenic gene activation [158]. Additional experimental studies on angiogenesis indicate that AEG-1 can increase HIF-1α levels in both endothelial and tumor cells. This supports the notion that AEG-1 contributes to maintaining and strengthening HIF-driven transcriptional responses, even in regions where oxygen availability fluctuates [108]. Current evidence suggests a potential positive feedback relationship between AEG-1and HIF-1α. On one hand, hypoxia and HIF-1α activity promotes AEG-1 upregulation. On the other hand, AEG-1 itself can elevate HIF-1α expression and enhance downstream transcriptional programs that support angiogenesis [159]. Indeed, direct HIF-1α binding to the AEG-1 promoter has been demonstrated [160]. HIF-1 activity is closely associated with shifts in cellular metabolism, particularly an increased reliance on glycolysis and other adaptations that enable survival in low-oxygen environments. These metabolic changes can also contribute indirectly to angiogenesis, for example through lactate accumulation and interactions with other signaling pathways, alongside the direct upregulation of VEGF-related genes. Broad analyses of HIF-1α function highlight its dual role in coordinating energy metabolism and promoting vascular development in solid tumors [161]. In the context of HCC, AEG-1 may help sustain this combined metabolic and angiogenic phenotype by preserving both HIF-1 transcriptional activity and PI3K/AKT signaling during hypoxic stress. Through this mechanism, AEG-1 supports continued expression of angiogenic factors while also enhancing the tumor’s ability to tolerate metabolic strain [131,159].

#### 5.2.2. Feedback Loop Between AEG-1 and HIF-1α

Evidence supports bidirectional regulatory interactions between AEG-1 and HIF-1α, although the level of mechanistic validation differs among cancer types. Studies outside HCC have demonstrated direct transcriptional activation of AEG-1 by HIF-1α through promoter binding, with positive-feedback signaling reported in ovarian and head-and-neck squamous cell carcinomas [160,162]. Conversely, AEG-1, through its established capacity to activate NF-κB, may promote HIF1A transcription, given that NF-κB p50/p65 has been shown to bind the HIF1A promoter and enhance its transcription in HCC cells [163]. In HCC specifically, hypoxia-associated NF-κB/HIF-1α signaling interplay is well established, but comparable evidence demonstrating direct HIF-1α interaction with the AEG-1 promoter in liver tumors remains limited. Therefore, it is more accurate to state that an AEG-1/HIF-1α feedback mechanism is probably conserved across malignancies, while direct promoter-level evidence is currently more robust in non-HCC settings.

#### 5.2.3. Impact on Metabolic Reprogramming That Favors Angiogenesis

The metabolic component of AEG-1 signaling in HCC is supported by emerging evidence, although it remains less comprehensively characterized than its established roles in NF-κB activation and VEGF-mediated angiogenesis. Early transcriptomic analyses in HCC models showed that AEG-1 overexpression upregulated pyruvate kinase, suggesting a shift toward enhanced glycolytic activity [28]. A subsequent study using glucose-deprivation conditions proposed that AEG-1 coordinates glycolytic regulation and angiogenic responses through HIF-1α-dependent modulation of glucokinase (GCK) and glucose transporter 2 (GLUT2), together with induction of VEGFC expression [164]. This framework is biologically plausible given the close relationship between hypoxia-driven metabolic adaptation and vascular signaling in HCC. However, the supporting mechanistic evidence remains comparatively limited and has not yet been extensively validated across independent studies. At present, the available data support the view that AEG-1 may integrate metabolic stress responses with pro-angiogenic signaling, although this aspect of AEG-1 biology is currently less firmly established than its documented effects on VEGF-family regulation and hypoxia-associated pathways.

### 5.3. Endothelial Activation and Vascular Remodeling

Experimental studies indicate that AEG-1 exerts direct effects on endothelial biology and angiogenic behavior. In endothelial cell models, including HUVECs, AEG-1 overexpression enhances invasive capacity and promotes capillary-like tube formation through activation of the PI3K/AKT signaling pathway [108]. Inhibition of AKT signaling markedly suppresses these pro-angiogenic effects, supporting a central role for PI3K/AKT-mediated signaling in AEG-1-driven endothelial activation. Additional functional studies demonstrate that Tie2 signaling is also required, as Tie2 silencing significantly reduces AEG-1-induced tube formation, while Tie2 and AKT signaling together appear to contribute to VEGF transcriptional regulation in this setting [108,109]. Collectively, these findings directly connect AEG-1 to endothelial morphogenesis and identify key signaling dependencies underlying its angiogenic effects. Although several of these observations were not generated exclusively in hepatic systems, corresponding evidence from HCC models supports their biological relevance in liver cancer. AEG-1-overexpressing hepatoma cells generate highly vascularized tumors in vivo, and conditioned media derived from AEG-1-overexpressing hepatocytes stimulate angiogenic responses in endothelial cells, supporting a paracrine mechanism of vascular activation [28,111]. In HCC, elevated AEG-1 expression has also been associated with increased production of angiogenic mediators including VEGF, PlGF, and FGFα, thereby promoting endothelial stimulation and neovascularization [28]. Evidence that AEG-1 interacts with both VEGF- and Tie2/Ang1-related pathways suggests that it may influence multiple stages of vascular remodeling, including both early angiogenic sprouting and subsequent vessel maturation [108]. This integrated activity may contribute to the structurally abnormal, unstable, and highly dynamic vasculature characteristic of aggressive HCC. Another evidence implicates AEG-1 in endothelial remodeling within the tumor microenvironment itself. Endothelial cells isolated from HCC tissues exhibit reduced miR-302c expression together with elevated AEG-1 levels, and increased endothelial AEG-1 promotes endothelial-to-mesenchymal transition (EndMT) [165]. Because EndMT contributes to pathological vascular remodeling, stromal conversion, and tumor-supportive microenvironment formation, these findings suggest that AEG-1 functions not only within malignant hepatocytes but also within the remodeled endothelial compartment of HCC. Overall, current evidence supports a model in which AEG-1 functions as an integrative regulator of HCC angiogenesis rather than acting through a single downstream pathway. Despite these advances, several important questions remain unresolved, including identification of the dominant angiogenic mediators regulated by AEG-1 in patient tumors, clarification of whether its primary vascular effects occur directly within endothelial cells or indirectly through tumor-derived paracrine signaling, and determination of the extent to which AEG-1 influences HIF-1α stability across different HCC etiologies.

## 6. AEG-1 and Microenvironmental Drivers of Angiogenesis

### Immune Modulation Contributing to Angiogenic Immunosuppression

Within HCC, the tumor microenvironment is shaped by a close interplay between vascular development and immune modulation. AEG-1 appears to function as an important coordinating factor in this context, linking signals that promote blood vessel formation with those that suppress effective immune responses. The role of VEGF in mediating these effects is particularly significant [166,167]. 

Sustained VEGF expression under AEG-1 regulation therefore contributes not only to aberrant vascular architecture but also to a microenvironment that discourages immune attack. Indeed, AEG-1 expression correlated with an immune population that represents immunosuppression [168]. AEG -driven activation of NF-κB further amplifies this effect by increasing the release of inflammatory mediators. These factors promote the recruitment of tumor-associated macrophages and bias their differentiation toward phenotypes that support tumor growth [169]. Once established, these macrophages secrete additional molecules—such as VEGF, matrix-remodeling enzymes, and TGF-β—that reinforce both angiogenesis and immune suppression, creating a self-reinforcing cycle. Indeed, deletion of AEG-1 from macrophages significantly abrogates their function and a myeloid-specific AEG-1 knockout mouse is markedly protected from MASH and HCC [99]. Low-oxygen conditions, which are common in HCC, intensify these processes [108,131,170]. Through stabilization of HIF-1α, AEG-1 enhances transcriptional programs that elevate VEGF levels while simultaneously supporting mechanisms that enable tumor cells to evade immune detection [108,131,170]. The combined effect is the development of a tumor environment marked by disorganized vasculature, weakened immune surveillance, and increased capacity for invasion and metastasis. Taken together, these findings support a model in which AEG-1 integrates multiple signaling networks to coordinate vascular and immune dynamics within HCC. By influencing cytokine production, immune cell behavior, and hypoxia-responsive pathways, it promotes the establishment of conditions that favor tumor persistence and limit therapeutic effectiveness.

## 7. AEG-1 and Therapeutic Resistance in Angiogenic HCC

Resistance to anti-angiogenic treatment may emerge through mechanisms other than renewed blood vessel formation. Studies using orthotopic HCC models have shown that tumors resistant to sorafenib can become more invasive and increasingly exploit pre-existing hepatic vasculature through vessel co-option instead of relying primarily on angiogenic sprouting [171]. Since vessel co-option depends on tumor cell invasion into surrounding tissue, this phenomenon may intersect with pathways regulated by AEG-1. Based on these observations, it is reasonable to hypothesize that tumors with elevated AEG-1 expression could adapt to anti-angiogenic therapy by increasing reliance on vessel co-option, although this proposed mechanism requires direct experimental validation.

Anti-angiogenic resistance in HCC has been increasingly linked to the activity of AEG-1, particularly in studies involving sorafenib, especially one study showing how sorafenib affects miR-375 signaling in hepatoma cells [172]. It was identified that sorafenib can increase expression of the tumor-suppressive microRNA miR-375 through ASH1 activity. miR-375 was found to inhibit angiogenesis partly by reducing PDGFC expression. Importantly, sorafenib-resistant cells displayed lower miR-375 levels, and restoring miR-375 expression helped recover sensitivity to sorafenib, partly through downregulation of AEG-1. These findings suggest that AEG-1 is involved not only in general drug resistance but also in mechanisms that allow tumors to maintain angiogenic activity despite treatment. Additional support for the role of AEG-1 in treatment resistance comes from studies examining hypoxia-related signaling. AEG-1 contributes to hypoxia-associated chemoresistance in HCC through activation of the PI3K/AKT/HIF-1α/MDR1 pathway [131]. The significance of AEG-1 may also extend beyond sorafenib-based treatment. Other therapies used in advanced HCC, including lenvatinib and the combination of atezolizumab with bevacizumab, similarly alter the tumor microenvironment by inducing vascular stress, hypoxia, and immune-related changes [22]. Because many of the downstream survival pathways are shared across these therapies, it is plausible that AEG-1 contributes to resistance in these settings as well. However, direct experimental evidence remains limited. 

**Figure 3:**
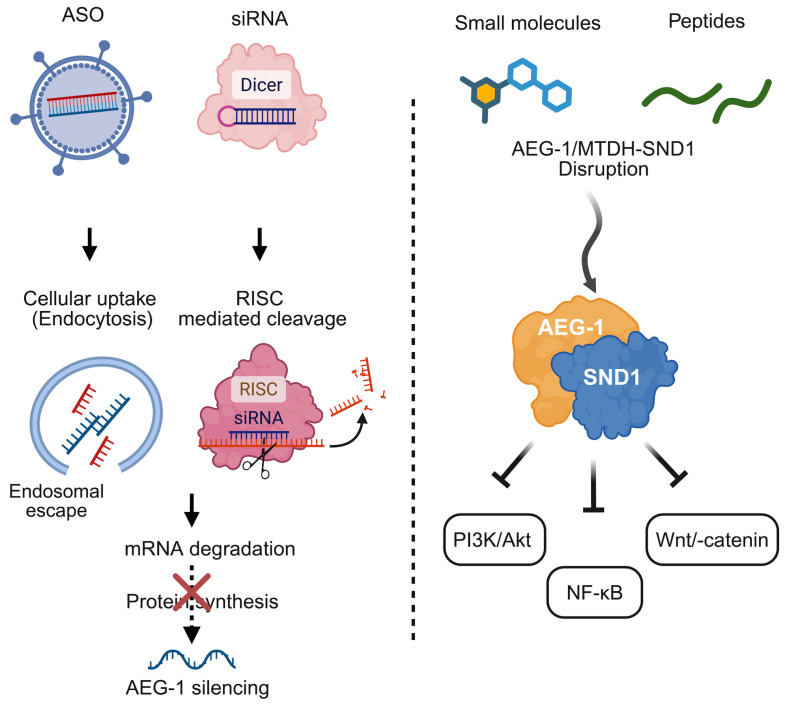
Therapeutic approaches targeting AEG-1/MTDH in cancer. The figure summarizes current strategies for targeting AEG-1, through gene-silencing and protein-interaction inhibition approaches. The left panel illustrates nucleic acid-based therapies, including ASOs and siRNAs. Following cellular uptake and endosomal escape, siRNAs are incorporated into the RISC, where they mediate sequence-specific cleavage and degradation of AEG-1 mRNA, resulting in reduced protein synthesis and effective AEG-1 silencing. The right panel depicts pharmacological strategies using small molecules or peptide-based inhibitors to disrupt the interaction between AEG-1/MTDH and SND1. Disruption of the AEG-1/MTDH–SND1 complex inhibits multiple oncogenic signaling pathways, including PI3K/Akt, NF-κB, and Wnt/β-catenin signaling, thereby suppressing tumor cell proliferation, survival, metastasis, and therapeutic resistance. Collectively, these approaches highlight the potential of AEG-1/MTDH-directed therapies as promising strategies for cancer treatment. AEG-1, astrocyte elevated gene-1; MTDH, metadherin; ASO, antisense oligonucleotide; siRNA, small interfering RNA; RISC, RNA-induced silencing complex; mRNA, messenger RNA; SND1, staphylococcal nuclease and Tudor domain-containing protein 1; PI3K, phosphoinositide 3-kinase; Akt, protein kinase B; NF-κB, nuclear factor kappa B; Wnt, Wingless/Integrated signaling pathway. Created with BioRender.

Targeted nanoparticle-mediated delivery of AEG-1 siRNA in combination with all-trans retinoic acid markedly suppressed growth of human HCC xenografts [173]. Although this work did not specifically investigate resistance to anti-angiogenic therapy, it established that AEG-1 suppression could produce measurable antitumor effects in vivo. Collectively, these studies support the feasibility of nucleic acid-based strategies for direct targeting of AEG-1 in liver cancer [174]. Small molecule inhibitors that interrupt AEG-1/SND1 interaction have been developed to inhibit in vivo growth of multiple tumors and synergize with anti-PD-1 therapy (Figure 3), thereby providing promising avenues of AEG-1 inhibition [125,126,127]. The efficacy of these inhibitors is yet to be evaluated in HCC. Although several therapeutic strategies may be developed from the current understanding of AEG-1 biology, significant limitations still need to be addressed. AEG-1 functions primarily as an intracellular scaffold for many proteins. Thus, inhibition of only one interaction, such as with SND1, may not be sufficient to efficiently eliminate its function, especially in inflammation-driven cancers where AEG-1-NF-κB interaction is pivotal. In addition, the biological activity of AEG-1 appears to vary across different cellular compartments, including hepatocytes and immune cells, complicating therapeutic selectivity. The efficient delivery of RNA-directed therapies within cirrhotic or fibrotic liver tissue also remains a significant translational obstacle. Furthermore, contemporary biomarker-driven studies have not yet clarified which patient populations would derive the greatest benefit from AEG-1-directed therapy. As such, therapeutic targeting of AEG-1 remains promising but is still largely confined to the preclinical setting. Table 1 provides a systematic categorization of the mechanistic evidence discussed above, distinguishing findings directly validated in HCC models from those supported primarily by other cancer models. 

## 8. Clinical and Prognostic Significance of AEG-1 

### 8.1. Correlation of AEG-1 Expression with Microvascular Invasion and Tumor Aggressiveness

AEG-1 has a well-established relationship with vascularly aggressive behavior in HCC, though the evidence base is considerably stronger for microvascular invasion and metastatic outcomes than for direct, standardized measurements of microvessel density (MVD). Analyzing AEG-1 expression using a tissue microarray comprising 109 HCC specimens and nine samples of adjacent non-malignant liver tissue unraveled detectable AEG-1 in 93% of HCC cases [28]. Additionally, its levels climbed progressively with both Barcelona Clinic Liver Cancer (BCLC) staging and histologic dedifferentiation. Immunohistochemical analysis of 323 surgically resected HCCs documented AEG-1 expression in just over half of the cases and was independently linked to microvascular invasion, satellite lesions, poor tumor differentiation, and advanced TNM staging (all *p* ≤ 0.007) [177]. In Cox multivariable regression analysis, AEG-1 retained independent prognostic significance for both endpoints. Examination of 288 primary HCCs found high AEG-1 expression in 47.9% of cases which correlated with higher Edmondson grade, microvascular invasion, higher AJCC T stage, elevated AFP, and younger age at presentation. On multivariable analysis, high AEG-1 independently predicted shorter disease-free survival after curative hepatectomy [178]. Another independent study in 89 HCC cases showed AEG-1 positivity in 57% of tumors with higher expression levels than those of paratumoral, cirrhotic, or normal liver controls [179]. High AEG-1 expression correlated with histologic grade, distant metastasis, and HBV infection, although associations with tumor size and vascular invasion did not reach significance in this smaller cohort. The accompanying meta-analysis, pooling seven HCC studies, confirmed that elevated AEG-1 robustly associated with poorer overall survival and with significantly higher odds of metastasis (OR 3.791; 95% CI 1.958–8.051). This variability across individual series underscores why aggregate evidence matters—even when single studies diverge on specific features, the adverse-prognosis signal holds [179]. Taken together, AEG-1 has a well-supported role as a marker of vascularly aggressive HCC, particularly for microvascular invasion, tumor recurrence, and distant spread. What remains conspicuously absent is a rigorous set of studies pairing AEG-1 protein expression directly with standardized intratumoral vessel counts, the kind obtained via CD34- or CD31-based MVD assays in resected specimens. Microvascular invasion and measured angiogenic density, while biologically related, are distinct tissue phenomena. AEG-1 can already be defended as a clinically meaningful marker of vascular aggressiveness in HCC; its standing as a quantitative indicator of tumor angiogenesis remains plausible but awaits more definitive validation.

### 8.2. Prognosis and Predictive Significance in Clinical HCC

The clinical prognostic data for AEG-1 in HCC stand on considerably firmer ground than its mechanistic vascular evidence. Across surgically resected cohorts, tissue overexpression of AEG-1 has repeatedly been linked to shorter recurrence-free and overall survival. Three studies provide the most compelling individual evidence. A study on 323 HCC patients found that those with high AEG-1 expression had significantly worse 1-, 3-, and 5-year overall survival compared to those with low expression, along with markedly higher cumulative recurrence rates [177]. In 288 HCC patients who underwent curative resection, elevated AEG-1 expression independently predicted reduced disease-free survival after surgery [178]. A striking feature of overall survival of 24.5% versus 89.2% and recurrence of 82.4% versus 50.0% in high versus low AEG-1 expressors, respectively, was unraveled by a smaller but carefully scored, blinded immunohistochemical series. Together, these findings reframe AEG-1 as a postoperative marker capable of meaningfully enriching risk stratification after resection [180]. The prognostic signal also holds up across different disease etiologies, with AEG-1 levels conferring survival disadvantages to both HBV- and HCV-driven HCC [28,181]. A meta-analysis synthesizing results across multiple published studies reported a pooled overall survival hazard ratio exceeding 2.0 for HCC, with high AEG-1 expression significantly associated with poor prognosis across gastrointestinal cancers including HCC (HR = 2.245, 95% CI: 1.620–3.113) [182]. This cross-cohort durability matters clinically: many candidate HCC biomarkers show early promise in single-center studies but lose their signal against more diverse disease backgrounds. From a methodological standpoint, these studies share recognizable strengths, such as use of authentic resected human tissue, established clinicopathologic endpoints, and in several cases semiquantitative immunohistochemistry performed under blinded pathologic review. Their limitations are equally familiar: retrospective design, single-center recruitment, variable assay protocols, non-standardized thresholds for defining "high" expression, and no benchmarking against contemporary prognostic tools such as transcriptomic subclassification or immune microenvironment characterization. AEG-1 is therefore best positioned at this stage as a candidate integrative prognostic biomarker, not one ready for independent clinical use. The predictive evidence whether AEG-1 status can identify which patients will benefit from a specific therapy remains far less developed. A true predictive biomarker requires human trial data showing differential treatment response by marker status, and that evidence does not yet exist for AEG-1 in HCC. 

### 8.3. Potential Utility as a Biomarker for Angiogenic Phenotype

The case for AEG-1 as a biomarker of angiogenically active HCC rests on converging evidence from three levels. Biological studies strongly position AEG-1 as a key regulator of angiogenesis. Clinicopathologically, high AEG-1 expression has consistently co-occurred with microvascular invasion, metastatic spread, and advanced disease stage across multiple resected cohorts. At the specimen level, both tissue-based and circulating assay approaches confirm that AEG-1-related signals are technically accessible in clinical material. Together, these observations make AEG-1 a biologically plausible surrogate for angiogenesis-driven tumor behavior in HCC, even though formal analytic validation as a biomarker remains incomplete. On the tissue-diagnostic front, immunohistochemistry in HCC, adjacent non-tumor liver, and dysplastic nodules for both AEG-1 and HCC marker glypican-3 (GPC-3) unraveled AEG-1 positivity in 92% of HCC cases with predominantly diffuse staining, in contrast to the more frequent focal pattern of GPC-3. AEG-1 alone showed high sensitivity but low specificity [183]. When the two markers were combined, diagnostic performance improved substantially reaching sensitivity of 94.6%, specificity of 89.5%, and overall accuracy of 90.5% suggesting AEG-1 adds meaningful discriminatory value as part of a multi-stain panel rather than as a standalone test.

Circulating biomarker work is still at an early stage but shows genuine promise. Measurement of serum AEG-1 mRNA alongside miR-497 in 60 HCC patients and 60 healthy controls, revealed that elevated AEG-1 expression correlated significantly with larger tumor size, multifocality, nodal and distant metastasis, vascular invasion, BCLC stage, and inferior overall survival [184]. Taken together, AEG-1 is a credible candidate biomarker for aggressive, angiogenically active HCC, particularly when that phenotype encompasses vascular invasion, metastatic competence, and an immunologically unfavorable microenvironment. Its most realistic near-term role is within multi-parameter models for example, combined tissue assessment with GPC-3 and endothelial markers such as CD34 or CD31 rather than as a standalone test. The principal barriers to clinical translation remain the lack of standardized expression-scoring thresholds, the scarcity of studies directly pairing AEG-1 levels with quantified intratumoral vessel density, and the absence of treatment-interaction analyses. Until those gaps are closed, AEG-1 is best characterized as a biologically well-grounded candidate awaiting the prospective, multi-center validation studies needed to move it toward routine clinical application.

## 9. Future Directions and Emerging Concepts 

### 9.1. Single-Cell and Spatial Transcriptomic Profiling of AEG-1-Positive Niches

Recent advances in single-cell RNA sequencing and spatial transcriptomics are beginning to reshape understanding of tumor heterogeneity in HCC, offering an opportunity to define AEG-1-positive cellular niches with greater precision than bulk transcriptomic analyses allow. This matters because AEG-1 is expressed not only in malignant hepatocytes but also influences endothelial, stromal, and immune cell populations within the tumor microenvironment [28,108]. Spatial transcriptomics analysis of WT and AEG-1-C75S mice livers unraveled that inhibition of AEG-1 palmitoylation selectively augments pro-inflammatory and MASH- and HCC-promoting pathways in periportal and pericentral hepatocytes, while mid-lobular zone showed inhibition in xenobiotic metabolism pathways [130]. This finding indicates that spatial expression and post-translational modification significantly modulates AEG-1 function. A key unanswered question is whether distinct AEG-1-high subpopulations preferentially localize to hypoxic, invasive, immune-suppressed, or highly vascularized tumor regions. Integrating single-cell transcriptomics with spatial proteomics and multiplex imaging could reveal coordinated expression programs spanning VEGF signaling, hypoxia-response pathways, and endothelial remodeling and might clarify whether AEG-1-driven angiogenesis differs across HCC etiologic subtypes, including HBV-, HCV-, MASH-, and alcohol-related disease. These approaches may ultimately identify angiogenic “hotspots” predictive of aggressive behavior, therapeutic resistance, or poor immunotherapy responsiveness.

### 9.2. Role of AEG-1 in Vessel Normalization Versus Aberrant Angiogenesis

Most existing evidence positions AEG-1 as a driver of structurally abnormal, dysfunctional tumor vasculature [108,111]. However, its relationship to vessel normalization and transient re-establishment of more functional vascular architecture under anti-angiogenic pressure remains poorly characterized. Because AEG-1 interacts with both VEGF-associated sprouting pathways and signaling networks linked to vascular stabilization [108], it may influence the balance between pathological angiogenesis and transient normalization states. Future experimental work should address whether selective modulation of AEG-1 signaling alters endothelial maturation, pericyte recruitment, perfusion efficiency, or vascular permeability and whether AEG-1 targeting could complement, rather than simply suppress, vascular remodeling in ways that enhance drug delivery and antitumor immunity.

### 9.3. Integration of Angiogenesis with Metabolic Reprogramming and Immune Evasion

One of the most important shifts in HCC biology is the recognition that angiogenesis, metabolic adaptation, and immune suppression are interconnected rather than independent hallmarks of tumor progression. AEG-1 is increasingly positioned as an integrative regulator linking these processes. Hypoxia activates both angiogenic signaling and metabolic reprogramming through HIF-dependent pathways [131], and AEG-1 appears capable of sustaining these adaptive responses via PI3K/AKT, NF-κB, and HIF-1α interactions [131]. Evidence also suggests that AEG-1 promotes glycolytic adaptation and tolerance to metabolic stress, thereby supporting tumor survival under hypoxic and nutrient-deprived conditions [162,175] while metabolic byproducts such as lactate may further reinforce angiogenesis through inflammatory signaling and sustained VEGF production [176]. On the immune side, AEG-1 contributes to an immunosuppressive microenvironment through regulation of inflammatory cytokines and NF-κB activity, potentially impairing dendritic-cell maturation, reducing cytotoxic T-cell function, and promoting recruitment of tumor-associated macrophages and myeloid-derived suppressor cells [185]. The convergence of these processes likely represents a coordinated tumor survival strategy. Future research integrating immunometabolism, angiogenesis biology, and spatial tumor ecology will be needed to clarify how AEG-1 orchestrates these programs and whether co-targeting them offers therapeutic advantages over single-pathway inhibition.

### 9.4. Translational Gaps and Unmet Clinical Needs

Despite growing mechanistic evidence supporting the role of AEG-1 in HCC angiogenesis, major translational challenges remain unresolved. One limitation is the lack of clinically validated biomarkers capable of reliably identifying patients with AEG-1-driven angiogenic phenotypes. Most current evidence derives from experimental systems or retrospective tissue analyses, whereas prospective clinical validation remains limited. Another important challenge is the absence of selective AEG-1-targeted therapies currently approved for clinical use. Although RNA interference strategies, peptide inhibitors, and small molecule inhibitors have shown encouraging preclinical activity, many obstacles remain regarding delivery efficiency, tumor specificity, pharmacokinetics, and toxicity. Moreover, AEG-1 presents intrinsic drug development challenges because it lacks a well-defined catalytic domain and exerts many of its oncogenic functions through protein–protein interactions and intracellular signaling networks. This limits the availability of conventional small-molecule targeting strategies and complicates therapeutic design. Furthermore, despite encouraging preclinical findings, no AEG-1-targeted therapy has yet advanced to clinical evaluation, highlighting the need for continued optimization of target engagement and delivery approaches. Additionally, the multifunctional nature of AEG-1 raises concerns that systemic inhibition could disrupt multiple physiological signaling networks beyond tumor tissue. Further complexity arises from the highly heterogeneous nature of HCC itself. Angiogenic dependence varies considerably among tumors, and compensatory signaling pathways may reduce the efficacy of single-agent therapies targeting AEG-1-associated networks. Consequently, future therapeutic development will likely require rational combination strategies incorporating angiogenesis inhibition, metabolic targeting, immune modulation, and microenvironment-directed therapies. Bridging these translational gaps will require integrated experimental models that better capture the biological complexity of HCC, including patient-derived organoids, spatially resolved tumor analyses, and immunocompetent in vivo systems. Such approaches may ultimately enable the development of precision-based therapeutic strategies targeting AEG-1-dependent angiogenic programs.

## 10. Conclusions

### 10.1. Summary of AEG-1 as a Central Orchestrator of Angiogenesis in HCC

**Figure 4:**
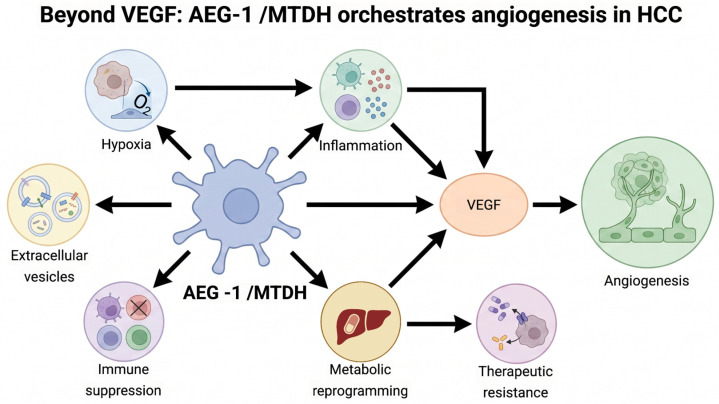
AEG-1/MTDH as a systems-level orchestrator of angiogenesis in HCC. AEG-1/MTDH acts as a central hub within the HCC tumor microenvironment, coordinating hypoxia, inflammation, immune suppression, metabolic reprogramming, and extracellular vesicle signaling to drive VEGF-dependent angiogenesis and therapeutic resistance. Arrows indicate directional regulatory relationships. AEG-1, astrocyte elevated gene-1; MTDH, metadherin; HCC, hepatocellular carcinoma; VEGF, vascular endothelial growth factor. Created with Biorender.

Accumulating evidence supports the concept that AEG-1 functions as a central systems-level regulator of angiogenesis in HCC rather than as a conventional single-pathway oncogene. Through interactions with PI3K/AKT, NF-κB, HIF-1α, Wnt/β-catenin, SND1, and inflammatory signaling networks, AEG-1 coordinates multiple biological processes that collectively sustain tumor vascularization, progression, and adaptation to environmental stress. Importantly, AEG-1 influences angiogenesis at several levels simultaneously, including transcriptional regulation of pro-angiogenic mediators, post-transcriptional and translational control of angiogenic signaling, endothelial remodeling, hypoxia adaptation, stromal communication, and immune modulation. This broad integrative activity distinguishes AEG-1 from many traditional angiogenic regulators and highlights its importance within the complex vascular biology of HCC. The integrative role of AEG-1/MTDH across these interconnected processes is illustrated in Figure 4.

### 10.2. Therapeutic Implications for Shifting Beyond VEGF-Centric Models

Current anti-angiogenic therapies in HCC largely focus on VEGF-centered signaling pathways. Although these approaches provide measurable clinical benefit, their efficacy is frequently limited by adaptive resistance, compensatory pathway activation, vessel co-option, immune suppression, and tumor heterogeneity. The emerging role of AEG-1 suggests that broader upstream regulatory networks may represent more effective therapeutic targets than isolated pathway inhibition alone. Because AEG-1 integrates multiple angiogenic and tumor-promoting signaling programs simultaneously, therapeutic strategies targeting AEG-1 or its associated signaling complexes may help overcome limitations associated with VEGF-only approaches. In particular, the ability of AEG-1 to coordinate inflammatory signaling, hypoxia adaptation, endothelial activation, metabolic stress responses, and immune suppression positions it as a potentially valuable therapeutic node within the HCC microenvironment.

### 10.3. Perspective on Advancing Next-Generation, Multi-Targeted Treatment Strategies

Future progress in HCC treatment will likely depend on the development of multi-dimensional therapeutic strategies capable of targeting the interconnected biological systems that sustain tumor progression. Rather than viewing angiogenesis, metabolism, immune evasion, and stromal remodeling as separate processes, emerging evidence supports the concept that these programs operate as highly integrated adaptive networks within HCC. In this context, AEG-1 represents a promising candidate for next-generation therapeutic development because of its ability to function at the intersection of these pathways. Future therapeutic strategies may combine AEG-1-directed approaches with immune checkpoint inhibitors, metabolic therapies, anti-fibrotic interventions, or vascular normalization strategies to improve treatment durability and overcome resistance. Ultimately, advancing the clinical translation of AEG-1 biology will require improved mechanistic understanding, biomarker-driven patient stratification, and carefully designed translational studies that integrate molecular profiling with functional tumor microenvironment analysis. Continued investigation into AEG-1-centered signaling networks may therefore help establish a new framework for understanding and therapeutically targeting angiogenesis in HCC.

## Figures and Tables

**Table 1:** Categorization of mechanistic evidence for AEG-1-driven angiogenic processes by cancer model origin. “HCC Evidence = Yes” indicates direct experimental evidence from HCC cell lines, HCC animal models, or human HCC tissue. “Other Cancer Evidence = Yes” indicates supporting evidence from non-HCC cancer models, generic endothelial models (e.g., HUVECs as primary model), or general tumor biology. Reference numbers correspond to the manuscript numbering.

Pathway/Mechanistic Process	Key Finding	HCC Evidence	Other Cancer Evidence	References
AEG-1/HIF-1α Feedback Loop				
AEG-1/HIF-1α axis	Hypoxia increases AEG-1 expression via PI3K/AKT/HIF-1α signaling	Yes	No	[131]
AEG-1/HIF-1α axis	AEG-1 contributes to hypoxia-driven chemoresistance via PI3K/AKT/HIF-1α/MDR1	Yes	No	[108,131]
AEG-1/HIF-1α axis	AEG-1 increases HIF-1α levels and pro-angiogenic endothelial responses	Yes	Yes	[108]
AEG-1/HIF-1α axis	HIF-1α directly binds AEG-1 promoter (transcriptional activation)	No	Yes	[160,162]
AEG-1/HIF-1α axis	NF-κB p50/p65 binds HIF1A promoter and enhances transcription	Yes	No	[163]
AEG-1/HIF-1α axis	AEG-1/HIF-1α positive feedback loop (bidirectional regulation)	Yes	Yes	[160,162,163]
**Endothelial Activation and Vascular Remodeling**				
Endothelial activation	AEG-1 overexpression enhances EC invasion and tube formation via PI3K/AKT	No	Yes	[108]
Endothelial activation	Tie2 signaling required for AEG-1-induced tube formation	No	Yes	[108,109]
Endothelial activation	AEG-1-overexpressing hepatoma cells generate highly vascularized tumors in vivo	Yes	No	[28,111]
Endothelial activation	Conditioned media from AEG-1-overexpressing hepatocytes stimulate angiogenic responses	Yes	No	[28,111]
Endothelial activation	AEG-1 upregulates VEGF, PlGF, and FGFα in HCC cells	Yes	No	[109]
Endothelial activation	Endothelial AEG-1 promotes EndMT via miR-302c downregulation	Yes	No	[165]
Endothelial activation	AEG-1 enhances Factor XII production via polysome loading	Yes	No	[111]
**Immune Regulation and Angiogenic Immunosuppression**				
Immune modulation	AEG-1 correlates with immunosuppressive cell populations	Yes	No	[168]
Immune modulation	AEG-1/NF-κB promotes TAM recruitment and pro-angiogenic cytokine release	Yes	Yes	[169]
Immune modulation	Myeloid-specific AEG-1 knockout protects from MASH and HCC	Yes	No	[99]
Immune modulation	AEG-1/HIF-1α enhances VEGF while supporting immune evasion	Yes	Yes	[108,131,170]
**Metabolic Reprogramming**				
Metabolic reprogramming	AEG-1 overexpression upregulates pyruvate kinase (glycolytic shift)	Yes	No	[28]
Metabolic reprogramming	AEG-1 coordinates glycolytic regulation and angiogenic responses via HIF-1α/GCK/GLUT2/VEGFC	Yes	Yes	[164]
Metabolic reprogramming	AEG-1 promotes glycolytic adaptation and tolerance to metabolic stress	No	Yes	[162,175]
Metabolic reprogramming	Lactate reinforces angiogenesis through inflammatory signaling and VEGF	No	Yes	[176]
**AEG-1 Oncogenic Partner Interactions**				
AEG-1/c-Myc	MYC directly regulates AEG-1 transcription; AEG-1 reinforces c-Myc	Yes	No	[135,145]
AEG-1/SND1	AEG-1-SND1 interaction in RISC promotes tumor suppressor mRNA degradation	Yes	No	[121,150,151,152]
AEG-1/SND1	Disruption of AEG-1-SND1 interaction abolishes tumorigenic capacity	No	Yes	[153]
AEG-1/Wnt/β-catenin	AEG-1 modulates Wnt components (LEF1, APC, CTBP2, GSK3β) to promote HCC	Yes	No	[28,154,155,156]
AEG-1/Wnt/β-catenin	Direct AEG-1/β-catenin interaction	No	Yes	[157]
**AEG-1 Regulation by PI3K/AKT and NF-κB**				
PI3K/AKT	Ha-ras induces AEG-1 via PI3K/c-Myc/E-box promoter binding	Yes	No	[135]
PI3K/AKT	AEG-1 activates PI3K/AKT (reciprocal activation loop)	Yes	No	[136]
NF-κB	TNF-α and IL-1β induce AEG-1 via NF-κB	Yes	Yes	[137,138,139]
NF-κB	AEG-1 interacts with p65/CBP to promote NF-κB nuclear signaling	Yes	Yes	[140,141]
NF-κB	AEG-1 interacts with TRAF2/RIPK1 facilitating NF-κB activation	Yes	Yes	[120,142]
**Therapeutic Targeting**				
Therapeutic resistance	Sorafenib/miR-375/AEG-1 axis in anti-angiogenic resistance	Yes	No	[172]
Therapeutic resistance	AEG-1 contributes to hypoxia-driven chemoresistance (PI3K/AKT/HIF-1α/MDR1)	Yes	No	[131]
Therapeutic resistance	AEG-1 siRNA + ATRA suppresses HCC xenograft growth	Yes	No	[173]
Therapeutic targeting	Small molecule AEG-1/SND1 inhibitors synergize with anti-PD-1	No	Yes	[125,126,127]
Therapeutic resistance	Vessel co-option as resistance mechanism (potential AEG-1 involvement)	Yes	Yes	[171]

*Abbreviations:* AEG-1, astrocyte elevated gene-1; AKT, protein kinase B; APC, adenomatous polyposis coli; ATRA, all-trans retinoic acid; CBP, CREB-binding protein; CTBP2, C-terminal binding protein 2; EC, endothelial cell; EndMT, endothelial-to-mesenchymal transition; FGFα, fibroblast growth factor alpha; GCK, glucokinase; GLUT2, glucose transporter 2; GSK3β, glycogen synthase kinase 3 beta; HCC, hepatocellular carcinoma; HIF-1α, hypoxia-inducible factor-1α; IL-1β, interleukin-1 beta; LEF1, lymphoid enhancer-binding factor 1; MASH, metabolic dysfunction-associated steatohepatitis; MDR1, multidrug resistance protein 1; miR-302c, microRNA-302c; miR-375, microRNA-375; NF-κB, nuclear factor kappa B; PD-1, programmed cell death protein 1; PI3K, phosphatidylinositol 3-kinase; PlGF, placental growth factor; RIPK1, receptor-interacting serine/threonine kinase 1; RISC, RNA-induced silencing complex; siRNA, small interfering RNA; SND1, staphylococcal nuclease and tudor domain-containing 1; TAM, tumor-associated macrophage; TNF-α, tumor necrosis factor alpha; TRAF2, TNF receptor-associated factor 2; VEGF, vascular endothelial growth factor; VEGFC, vascular endothelial growth factor C.

## Data Availability

No new data were created or analyzed in this study. Data sharing is not applicable to this article.

## Abbreviations

The following abbreviations are used in this manuscript:

**AEG-1**	Astrocyte elevated gene-1
**AFP**	Alpha-fetoprotein
**AJCC**	American Joint Committee on Cancer
**AKT**	Protein kinase B
**Ang-1**	Angiopoietin-1
**Ang-2**	Angiopoietin-2
**APC**	Adenomatous polyposis coli
**BCLC**	Barcelona Clinic Liver Cancer
**CBP**	CREB-binding protein
**CD31**	Cluster of differentiation 31 (endothelial marker)
**CD34**	Cluster of differentiation 34 (endothelial marker)
**cDNA**	Complementary DNA
**CI**	Confidence interval
**CTBP2**	C-terminal binding protein 2
**CXCL12**	C-X-C motif chemokine ligand 12
**CXCL16**	C-X-C motif chemokine ligand 16
**CXCR4**	C-X-C chemokine receptor type 4
**DEN**	N-nitrosodiethylamine
**DLL4**	Delta-like canonical Notch ligand 4
**DNA**	Deoxyribonucleic acid
**EC**	Endothelial cell
**ECM**	Extracellular matrix
**EndMT**	Endothelial-to-mesenchymal transition
**EPO**	Erythropoietin
**ER**	Endoplasmic reticulum
**ERK**	Extracellular signal-regulated kinase
**FGF**	Fibroblast growth factor
**FGFR1**	Fibroblast growth factor receptor 1
**GCK**	Glucokinase
**GLUT2**	Glucose transporter 2
**GPC-3**	Glypican-3
**GSK3β**	Glycogen synthase kinase 3 beta
**HBV**	Hepatitis B virus
**HCC**	Hepatocellular carcinoma
**HCV**	Hepatitis C virus
**HGF**	Hepatocyte growth factor
**HIF**	Hypoxia-inducible factor
**HIF-1α**	Hypoxia-inducible factor-1 alpha
**HIF-2α**	Hypoxia-inducible factor-2 alpha
**HIV-1**	Human immunodeficiency virus type 1
**HR**	Hazard ratio
**HSC**	Hepatic stellate cell
**HUVEC**	Human umbilical vein endothelial cell
**ICI**	Immune checkpoint inhibitor
**IKKβ**	Inhibitor of nuclear factor kappa B kinase subunit beta
**IL-1**	Interleukin-1
**IL-8**	Interleukin-8
**KC**	Kupffer cell
**LEF1**	Lymphoid enhancer-binding factor 1
**LYRIC**	Lysine-rich CEACAM-1 co-isolated protein
**MAFLD**	Metabolic dysfunction-associated fatty liver disease
**MAPK**	Mitogen-activated protein kinase
**MASH**	Metabolic dysfunction-associated steatohepatitis
**MDR-1**	Multidrug resistance protein 1
**miRNA**	MicroRNA
**MMP**	Matrix metalloproteinase
**MTDH**	Metadherin
**MVD**	Microvessel density
**MYC**	MYC proto-oncogene
**NF-κB**	Nuclear factor kappa-light-chain-enhancer of activated B cells
**NLS**	Nuclear localization signal
**OR**	Odds ratio
**PAF**	Platelet-activating factor
**PDGF**	Platelet-derived growth factor
**PDGF-β**	Platelet-derived growth factor beta
**PDGFC**	Platelet-derived growth factor C
**PHFA**	Primary human fetal astrocyte
**PI3K**	Phosphatidylinositol 3-kinase
**PlGF**	Placental growth factor
**PLZF**	Promyelocytic leukemia zinc finger (ZBTB16)
**PTEN**	Phosphatase and tensin homolog
**RaSH**	Rapid subtraction hybridization
**RIPK1**	Receptor-interacting serine/threonine kinase 1
**RISC**	RNA-induced silencing complex
**RNA**	Ribonucleic acid
**ROS**	Reactive oxygen species
**RXR**	Retinoid X receptor
**siRNA**	Small interfering RNA
**SND1**	Staphylococcal nuclease and tudor domain-containing 1
**STAT3**	Signal transducer and activator of transcription 3
**TCGA**	The Cancer Genome Atlas
**TGF**	Transforming growth factor
**TKI**	Tyrosine kinase inhibitor
**TMD**	Transmembrane domain
**TME**	Tumor microenvironment
**TNF**	Tumor necrosis factor
**TNM**	Tumor-Node-Metastasis staging system
**TRAF2**	TNF receptor-associated factor 2
**TSP-1**	Thrombospondin-1
**VEGF**	Vascular endothelial growth factor
**VEGF-A/B/C/D/E**	Vascular endothelial growth factor isoforms A–E
**VEGFR1/2/3**	Vascular endothelial growth factor receptors 1, 2, and 3
**ZBTB16**	Zinc finger and BTB domain containing 16
**ZDHHC6**	Zinc finger DHHC-type palmitoyltransferase 6
